# Adiposity and feeding practices in the first two years of life among toddlers in Guadalajara, Mexico

**DOI:** 10.1186/s12887-023-03877-7

**Published:** 2023-02-04

**Authors:** Citlalli Álvarez-Zaragoza, Edgar M. Vásquez-Garibay, Carmen Alicia Sánchez-Ramírez

**Affiliations:** 1 Institute of Human Nutrition, University of Guadalajara, Guadalajara, México; 2 University of Colima, Colima, México

**Keywords:** Toddlers, Adiposity, Breastfeeding, Complementary feeding

## Abstract

**Background:**

Feeding practices in the first two years of life have a direct impact on nutritional status and adiposity. The purpose of this study was to identify the differences in feeding practices during the first two years of life by sex and type of feeding in the first semester of postnatal life and their relationships with adiposity in toddlers.

**Methods:**

An analytical cross-sectional study that included 150 toddlers aged 12 to 24 months who were healthy, full-term, and had adequate weight for their gestational ages, was conducted at the New Civil Hospital and at a private practice in Guadalajara. Body compositions were obtained by bioelectrical impedance (BIA) measurements, and a modified questionnaire was used. Then, the parents completed two 24-h dietary recalls. In addition to the descriptive statistics, ANOVA, Kruskal–Wallis and Mann–Whitney U tests were used in the contrast analysis of the quantitative variables. To analyze the qualitative variables, we used X^2^ tests. Afterward, linear regression tests were conducted to identify the relationships between adiposity and feeding practices during the first two years.

**Results:**

There were direct relationships between adiposity and duration of full breastfeeding (*r* = 0.610, *p* = 0.021), age of introduction of ultra-processed products (*r* = 0.311, *p* = 0.011), sugar (*r* = 0.186; *p* = 0.024) and age at which eggs were introduced (*r* = -0.202; *p* = 0.016).

**Conclusions:**

Adiposity was related to feeding practices in the first two years of life in toddlers.

## Background

Childhood obesity has become a major public health concern because of the cardiometabolic and noncardiometabolic consequences of obesity that significantly increase the risk of morbidity and mortality not only during childhood but also in adulthood [1]. Its high prevalence in childhood has become a major concern because obese children are at great risk of continuing to be obese in adulthood [2]. In Mexico, 8% of children aged 0 to 4 years are overweight and obeseand 22% are at risk of becoming overweight [3].

The first thousand days of life, from conception through the first two years of postnatal life, represent the most important period for human growth and development [4], and this period is considered a crucial window to preventing overweight or obesity and its adverse consequences [5, 6]. Consequently, it is desirable that interventions to prevent these conditions be carried out during this 1000-day period and that they cover all factors related to obesity risk [2]. Therefore, body composition measurements at 12–24 months of age are important since there are changes in muscle and bone tissue distributions and in the storage of fat reserves according to age and sex [7, 8].

Feeding practices in the first two years of life refer to breast or human milk substitutes feeding in the first semester of postnatal life and to complementary feeding (CF) from six to 24 months, which have direct impacts on nutritional status and body composition. It has been reported that growth patterns and changes in adiposity are different in breastfed infants vs. with those fed human milk substitutes (HMS) [9–11]. Infants who receive breastfeeding (BF) have higher fat mass at 2, 4 and 6 months of age [12, 13]. This is likely to protect infants by providing higher levels of energy stores during the first six months of life [11], which could be related to protection against obesity development in later stages of life [14, 15]. In contrast, HMS-fed infants gain weight more rapidly in the second postnatal year than infants receiving BF, resulting in higher BMI (body mass index) [11]. These differences in weight gain trends could contribute to an increased obesity risk in HMS-fed infants [16].

In relation to CF, specifically the age of solid food introduction, it has been reported that an early introduction (before 17 weeks of life) increases the risk of obesity [17, 18], although this assertion is not shared by other authors [14, 19]. On the other hand, CF practices from 12 to 24 months represent a critical period due to growth deceleration, and consequently, energy and nutrient intakes must be optimal to guarantee an adequate nutritional status while avoiding excess energy and/or nutrient intakes [18]. In addition, integrating toddlers into family diets requires special vigilance to avoid the consumption of ultra-processed foods and sugary drinks during this period because this could affect the development of appropriate eating habits and consequently lead to a deficit or excess of adiposity and eventually affect health status [16–18].

## Methods

The aim of this study was to identify the differences in feeding practices in the first two years of life by sex and type of feeding in the first semester of postnatal life and their relationships with adiposity among toddlers in Guadalajara, Mexico. In a cross-sectional analytical study, toddlers who were between 12 and 24 months of age in Guadalajara, Mexico from January 2020 to January 2021 were included. The required sample size was calculated as 145 infants with an alpha level of 0.01, power of 0.80, a correlation coefficient of 0.3 [20], and 20% of eliminations (for probable losses or when it was not possible to conduct Bioelectrical Impedance (BIA) measurements in some toddlers) using a non-probabilistic sampling system for convenience and that focused on the concentration sites.

### Recruitment of participants

We attended outpatient pediatric consultations at the New Civil Hospital “Dr. Juan I. Menchaca”, and at the private practices of three pediatric clinics in Guadalajara, Mexico. The purpose was to recruit toddlers between 12 and 24 months who attended a healthy-child control program and met the inclusion criteria (e.g., healthy, full-term, and birth weight appropriate for gestational age) [21]. Subsequently, we invited the mothers or legally responsible persons to participate in the study; if they chose to participate, we requested signatures indicating informed consent, and we continued with evaluations of the toddlers involved.

One hundred fifty toddlers were included, four of whom were eliminated because it was not possible to conduct BIA measurements because these toddlers removed the electrodes, and the analysis could not be continued.

### Techniques and instruments for obtaining information

BIA was performed on all participants by the same researcher using the same analyzer (Quadscan 4000, Body stat® Isla de Man) using 50-kHz measurements as follows: two electrodes were placed on the back of the right hand (at the wrist and metacarpus) and two on the dorsum of the foot (at the metatarsus and ankle). A red cable was connected to the distal electrode, and a black cable was connected to the proximal electrode. The measurements were obtained after two hours of fasting, and the state of hydration of each toddler on the previous day or factors that could affect this state (e.g., vomiting or diarrhea) were monitored with the participant in a supine position on a covered mattress free of electrically conductive materials and at room temperature [22, 23]. Adiposity values (kg and %) were obtained directly at the end of the BIA analyses; subsequently, the Z scores were calculated using kg values of adiposity and using Butte’s reference values (mean and standard deviation). To evaluate the type of feeding during the first semester of postnatal life and the introduction of CF, a questionnaire adapted from Perales [20] was used. The BF criteria were as follows: Full breastfeeding (FBF) was defined as when the infant was exclusively breastfed or predominantly breastfed during the first months of postnatal life. Exclusive breastfeeding (EBF) was defined as FBF with the administration of only additional drops or syrups of vitamins, minerals or medicines. Predominant breastfeeding (PrBF) consisted of FBF along with consumption of water, water-based drinks, juice and oral hydration, ritual liquids (herbal teas), drops or syrups of vitamins, minerals or medications. Partial breastfeeding (PBF) was considered to occur when, in addition to breastfeeding, babies received an HMS based on cow's milk or milk of vegetable origin at least once a day during the first six months of postnatal life [24].

Two 24-h dietary recalls were obtained (one during the week and the other on a weekend) by the same trained researcher [25]. The mother or caregiver of each toddler was questioned about all foods and beverages consumed during the previous 24-h period. The amount of each food or ingredient consumed was estimated using household measurements (including spoons, cups, slices, and handfuls) and food replicates. Subsequently, the energy, macronutrient and micronutrient intakes were calculated using Nutrimind software® (CDMX, Mexico). Information for both surveys was obtained directly through interviews with the mother or person in charge of feeding the toddler.

### Data processing

Data preparation was carried out using Microsoft Excel® for organization, error detection and to eliminate repetitive or incomplete information, and the data was later analyzed with SPSS® software (Southfield, MI).

### Statistical analysis

Descriptive quantitative statistics were obtained (mean and standard deviation or median and range) considering the normality of the data distributions that were obtained with the Kolmogorov–Smirnov tests. ANOVA,> Kruskal–Wallis and Mann–Whitney U tests were used for the contrast analysis of quantitative variables. For the qualitative variables, we used frequency and percent, and for comparisons between two groups, we used chi square (X^2^) analyses. Afterward, linear regression tests were conducted to identify the relationships between adiposity and feeding practices during the first two years.

A *p*-value < 0.05 was considered to be significant. Statistical analyses were performed with SPSS® software, version 22.

### Ethical considerations

Informed consent was obtained from all persons who were legally responsible for the participants, and the research protocol was approved by the ethics and research committee of the New Civil Hospital of Guadalajara “Dr. Juan I. Menchaca”, with registration number 0326/19 HCJIM. The approval date was November 14, 2019.

## Results

### General characteristics

Among all toddlers (*n* = 146), 51% were female and 49% were male; their average age was 17 ± 3 months. Their median weight was 10.0 kg (7.0–15.0 kg), and their median length was 78 cm (69–94 cm) (data not shown in the tables).

### Feeding in the first semester of postnatal life

Among all toddlers, 59% (*n* = 86) received full breastfeeding (FBF), 34.2% (*n* = 50) received PBF, and only 6.8% (*n* = 10) consumed HMS; most of them received a formula designed for infants. There were no differences by sex. A total of 53.4% of toddlers received exclusive breastfeeding (EBF), and even though the frequencies were higher in females than in males (57.3% vs. 49.3%, respectively), there were no significant differences. The median duration of FBF (EBF and PrBF) was six months, and for PBF was four months, with no significant differences by sex (data are not shown in the tables).

### Introduction of complementary foods

In the total studied population, the average age at which complementary foods were introduced was 5.8 ± 0.7 months. The average age at which fruits were introduced was 6 ± 1.1 months, vegetables at 6.1 ± 1.1 months, cereals at 6.9 ± 1.5 months and fortified cereals at 6.7 ± 1.6 months of age. Legumes and red meat were introduced at eight months, and chicken, fish and eggs were introduced between eight and nine months. The age at which added sugars, ultra-processed foods and natural fruit juices were introduced occurred before twelve months of age. The average age of introduction of soft drinks was 14 ± 4 months, and for processed juices, it was 12 ± 3 months. By sex, there were no significant differences in the ages at which complementary foods were introduced. (Data are not shown in the tables).

Toddlers who consumed HMS received complementary foods later than those who received full breastfeeding (FBF) or PBF. Post hoc tests showed that the age of introduction of oilseeds was earlier among those who received FBF vs. PBF. Likewise, toddlers who consumed FBF received vegetables, red meat, chicken, and oilseeds earlier than those who received HMS. Among toddlers who received PBF, vegetables, red meat, chicken, and oilseeds were introduced at earlier ages than among those who received HMS (Table 1).

**Table 1:** Age of introduction of complementary foods by type of feeding in the first semester of postnatal life

Age of food introduction (months)	FBF		PBF		HMS		*p*
	***n***	**X ± SD**	***n***	**X ± SD**	***n***	**X ± SD**	
Starting age of CF	86	5.9 ± 0.5	50	5.6 ± 0.9^A^	10	6.2 ± 1.0^A^	0.044
Fruits	86	6.1 ± 1.0	50	5.7 ± 1.1^B^	10	6.7 ± 2.0^B^	0.035
Vegetables	86	6.1 ± 0.9^C^	50	5.9 ± 0.6^D^	10	7.6 ± 2.6^C,D^	0.000
Cereals	86	6.8 ± 1.4	50	6.8 ± 1.6	10	7.7 ± 2.0	0.239
Fortified cereals	59	6.5 ± 1.0	34	7.0 ± 2.4	7	6.6 ± 1.1	0.462
Legumes	86	7.8 ± 2.0	49	8.3 ± 2.5	10	9.2 ± 2.8	0.127
Red meat	85	8.3 ± 2.7^D,E^	50	8.5 ± 2.3^D,F^	9	11.7 ± 3.3^E,F^	0.001
Chicken	85	7.5 ± 2.1^G^	50	7.8 ± 2.1^H^	10	9.8 ± 3.9^G,H^	0.012
Egg	86	8.3 ± 2.5	47	9.3 ± 3.2	10	9.9 ± 2.8	0.068
Fish and shellfish	78	9.6 ± 3.0	40	9.9 ± 2.5	6	12.5 ± 4.5	0.070
Cheese	78	10.6 ± 2.4	47	10.8 ± 2.9	9	11.8 ± 3.3	0.425
Oilseeds	71	9 ± 3.4^I,J^	40	10.8 ± 4.6^I^	5	15.2 ± 5.0^ J^	0.001
Fats	86	7.5 ± 2.6^ K^	49	8.4 ± 2.8^ K^	9	9.6 ± 2.7^ K^	0.033
Whole cow's milk	67	12.4 ± 1.9	38	13.3 ± 3.4	9	12.8 ± 3.7	0.231
Yogurt without added sugar	62	10.7 ± 2.2	29	9.9 ± 2.9	2	10.5 ± 2.1	0.364
Yogurt with added sugar	32	11.1 ± 3.6	26	12.7 ± 4.8	8	10.1 ± 3.8	0.237
Natural fruit juice	57	10.9 ± 3.6	34	11.1 ± 3.8	6	11.0 ± 3.6	0.974
Processed juice	33	12.3 ± 3.9	25	13.0 ± 3.6	8	10.5 ± 3.6	0.245
Soft drink	15	1.7 ± 4.5	12	16.4 ± 3.3	8	14.9 ± 5.6	0.287
Cookies or sweet bread	71	10.7 ± 3.1	49	10.1 ± 3.2	10	10.3 ± 3.5	0.639
Added sugars	63	11.8 ± 2.6	37	12.3 ± 3.7		10.7 ± 5.5	0.424

Statistics: ANOVA. *CF* Complementary feeding, *FBF* Full BF, *PBF* Partial breastfeeding, *HMS* Human milk substitute. n: number of participants, X: average, *SD* Standard deviation, and *NS* Nonsignificant

Post hoc Bonferroni: Starting age of CF: ^A^ (*p* = 0.072), Fruits: ^B^ (*p* = 0.062); Vegetables: ^C (^*p* < 0.001), D(*p* < 0.001), Red meat:

^E^ (*p* = 0.001); ^F^(*p* = 0.003), Chicken: ^G^ (*p* = 0.008),^H^*(p* = 0.028), Oilseeds: ^I^(*p* = 0.041); ^J^(*p* = 0.002) and fats: ^K^(NS)

### Complementary feeding from 12 to 24 months

At total of 61.7% of toddlers consumed sugary drinks; the most frequently consumed were juices (60%), soft drinks (13%) and fruit waters (27%). The consumption frequency was three times a week, and the median consumption amount was 55 (0–120) mL/d. Ninety-one percent of toddlers consumed ultra-processed foods, and those most frequently consumed were cookies (75%), sweet bread (22%) and sweets (8%). The consumption frequency was three times a week, and the median consumption amount was 1 (0–1) portion per day. There were no differences by sex or by type of feeding during the first semester of postnatal life. (Data are not shown in the tables).

Regarding intake by toddlers, analyzing the 24-dietary recall data during the week showed that the median energy consumption was 923 kcal/d. Regarding the macronutrient distributions, the median were 53% carbohydrates (CH), 14% proteins and 32% fats. Toddlers consumed 7 g/d of saturated fat (3–11 g/d) and 157 mg/d of cholesterol (44–305 mg/d), the sodium intake was 367 mg/d (228–589 mg/d) and the intake of added sugars was 8.5 g/d (0–24 g/d), all of which are expressed as median and interquartile range values. There were no significant differences by sex in energy, macro- or micronutrient intake for each weekday. On weekends, the 24 dietary recall data points showed that males had higher CH consumption than females (51 g vs. 49 g; *p* = 0.039), protein (34 g vs. 30 g, *p* = 0.032) and fiber (11 g vs. 7 g, *p* = 0.002). (Data are not shown in the tables).

After dividing the toddlers by the type of feeding they received in the first semester of postnatal life, toddlers who received HMS had higher intakes of energy, saturated fat and CH than those who received FBF or PBF (Table 2).

**Table 2:** Energy, macronutrient and micronutrient intakes by type of feeding in the first semester of postnatal life among toddlers aged 12–24 months

Variables	FBF (*n* = 86)		PBF (*n* = 50)		HMS (*n* = 10)		*p*
	**Median**	**(Q 25, Q 75)**	**Median**	**(Q 25, Q75)**	**Median**	**(Q 25,Q75)**	
Energy (kcal/d)	763	(577,1049)^A,B^	969	(670, 1143)^A^	1034	(720, 1589)^B^	0.027
Carbohydrates (%)	50	(46, 54)	50	(43, 55)	54	(50, 57)	0.242
Proteins (%)	15	(13, 20)	15	(13, 18)	13	(12, 15)	0.111
Fats (%)	34	(27, 38)	34	(28, 39)	32	(29, 35)	0.892
Proteins (g/d)	30	(24, 41)	33	(29, 45)	40	(24, 49)	0.210
Fats (g/d)	29	(20, 42)	35	(26, 46)	35	(25, 57)	0.124
Saturated fats (g/d)	4	(3, 8)^C,D^	8	(3, 14)^C^	13	(6, 19)^D^	0.001
Carbohydrates (g/d)	99	(70,125)^E,F^	119	(80, 146)^E^	158	(94, 217)^F^	0.019
Fiber (g/d)	8	(5, 12)	8	(5, 13)	12	(7, 16)	0.441
Cholesterol (mg/d)	145	(39, 257)	136	(63, 342)	65	(13, 275)	0.503
Sodium (mg/d)	382	(200, 634)	428	(291, 641)	573	(435, 992)	0.05
Added sugar (g/d)	7	(0, 25)	5	(0, 15)	15	(7, 37)	0.242
Added sugar (kcal/d)	9	(0, 20)	12	(0, 20)	9	(3, 18)	0.959

Statistics: Kruskal–Wallis, *Q* Quartile, *FBF* Full breastfeeding, *PBF* Partial breastfeeding, *HMS* Human milk substitute, *kcal/d* Kcal per day, and *g/d* Grams per day. Energy: ^A^ (*p* = 0.036), energy ^B^ (*p* = 0.041), saturated fats ^C^ (*p* = 0.002), saturated fats ^D^ (*p* = 0.007), CH: ^E^ (*p* = 0.048), and CH ^F^ (*p* = 0.028)

### Adiposity indicators obtained by BIA measurements from 12 to 24 months

Girls had lower adiposity Z scores than boys (Table 3). The adiposity indicators based on the type of feeding toddlers received in the first semester of postnatal life did not show any significant differences among those who received FBF, PBF or HMS. (Data are not shown in the tables).

**Table 3:** Adiposity indicators obtained from BIA measurements in the entire studied population and by sex

Variables	Total (*n* = 146)		Girls (*n* = 75)		Boys (*n* = 71)		*p*
	**Median**	**(Q 25, Q75)**	**Median**	**(Q 25, Q75)**	**Median**	**(Q 25, Q75)**	
Fat (kg)	2.6	(1.9,3.2)	2.5	(1.6, 3.1)	2.7	(2.0, 3.2)	0.224
Fat (%)	26.3	(19.3,30.8)	26.7	(18.6, 32.4)	26.3	(20.0, 29.8)	0.735
Fat (Z -score)	-0.25	(-1.44, 0.58)	-0.59	(-2.24, 0.68)	-0.10	(-0.95, 0.58)	0.05

Statistics: Mann–Whitney U test; *Q* Quartile

### Relationship between adiposity and feeding practices in the first two years of life

In the entire studied population, there was a direct relation between adiposity (%) and duration of FBF (*r* = 0.610, *R*^2^ = 0.372, *p* = 0.021) with adiposity expressed as Z scores and duration of FBF (r = 0.560, *R*^2^ = 0.313, *p* = 0.037). There were no significant relationships between adiposity and PBF duration, onset age of HMS, or feeding duration with HMS. In girls, there was a significant direct relation between adiposity (%) and FBF duration (*r* = 0.841, *R*^2^ = 0.707, *p* = 0.009). For boys, there were no significant relationships. (Data are not shown in the tables).

Table 4 shows the linear regression results between adiposity and introduction of complementary foods in the entire studied population and by sex. There were different linear regression trends depending on the way in which the adiposity was expressed (e.g., kg, % or Z score). When the adiposity was expressed in %, there were more significant relations among females.

**Table 4:** Linear regression results between adiposity (dependent variable) and age of introduction of complementary foods (independent variables) in the entire studied population and by sex

Dependent variable	Age of introduction (months)	r	R^2^	*p*
**Total population**				
Adiposity (%)	Egg	-0.202	0.040	0.016
Adiposity (kg)	Yogurt with added sugars	0.311	0.09	0.011
Adiposity (Z score)	CF	0.162	0.026	0.05
	Egg	0.165	0.027	0.048
	Yogurt with added sugars	0.263	0.069	0.033
Adiposity (%)	Females			
	CF	0.224	0.050	0.05
	Egg	-0.261	0.068	0.025
	Fish and seafood	-0.250	0.062	0.048
	Cheese	-0.237	0.056	0.048
	Oilseeds	-0.307	0.094	0.016
Adiposity (Z score)	Males			
	Yogurt with added sugars	0.387	0.149	0.046

r: correlation coefficient, R^2^: adjusted coefficient of determination, *CF* Complementary feeding

In the entire studied population and by sex, there were no significant relationships between adiposity and energy and nutrient intake on weekdays. However, when analyzing weekend days, we found that there were direct relations between adiposity (Z scores) and sugar (kcal) (*r* = 0.186; *R*^2^ = 0.03, *p* = 0.024) in the entire studied population and in females (*r* = 0.233; *R*^2^ = 0.05 *p* = 0.044). (Data are not shown in the tables).

We divided the toddlers by the type of feeding they received in the first semester of postnatal life; among those who received FBF or PBF, we did not find any significant relationships between adiposity (%, kg or Z score) and energy or nutrient intake. However, among those who received HMS, we found various relationships between adiposity (kg) and consumption of sugar-sweetened beverages, % of fats and % of CH. (Table 5).

**Table 5:** Linear regression results between adiposity (dependent variable) and current nutrient intakes (independent variable) in toddlers who received HMS in the first semester of postnatal life

Dependent variable	Intake of nutrients	r	R^2^	*p*
**Adiposity (Z- score)**	Sugary drinks intake (days/week)	0.741	0.549	0.022
	% CH	0.695	0.483	0.026
	% fats	0.732	0.535	0.016
	Saturated fats (g/day)	0.614	0.376	0.05
	Added sugars (kcal/day)	0.629	0.395	0.05

r: correlation coefficient, R^2^: adjusted coefficient of determination, and *CH* Carbohydrates, *g* Grams

## Discussion

### Feeding in the first semester of postnatal life

In the present study, 53.4% of infants received EBF in the first six months of life, which is a higher frequency than that reported for Mexico (28.6%) [3]. The median duration of EBF and PrBF were six months, which adheres to the recommendations of the WHO [26], and the frequency of EBF adheres to the Global Nutrition Targets 2025 recommendation to increase the EBF rate by 50% during the first six months [27].

### Introduction of complementary foods

The age of CF introduction adheres to the recommended starting age of months [18, 19], and this is consistent with data obtained for Mexican toddlers by Perales [20]. When analyzing the order of introduction of food groups, it was observed that fruits, vegetables, cereals and fortified cereals were introduced at six months, which adheres to the recommendation that this group of foods should be started at the beginning of CF [28, 29]. Nevertheless, sugary drinks and ultra-processed foods were introduced before 12 and 24 months, respectively. These findings are consistent with other studies of Mexican toddlers [20, 30], and they represent a focus of attention regarding the important deviations found in the feeding habits of toddlers [26].

An interesting finding was that toddlers who received FBF (exclusive and predominant) and PBF in the first semester of postnatal life received vegetables, red meat, chicken, and oilseeds at younger ages than those who received HMS. BF is associated with healthier dietary behaviors, and mothers who offer breastfeeding are more likely to offer healthy foods [17, 31]. We did not find any significant differences between those who received FBF vs. PBF, so it seems that the effect of breastfeeding in any of its forms is a strong incentive for adequate CF.

### Complementary feeding from 12 to 24 months

The importance of diet from six to 24 months has been described because this period corresponds to the formation of eating habits that contribute to overall health [32]. A finding that could be a focus of attention was the consumption of ultra-processed foods from 12 to 24 months, since 91% of infants consumed one portion per day approximately three times a week. This result is consistent with the increased consumption of ultra-processed foods among Mexican children and agrees with data obtained by Perales [20] for Mexican toddlers. Consumption of ultra-processed foods before the age of two years can lead to malnutrition, as they have a higher energy density, excessive amounts of fat and saturated fat, higher concentrations of sugar and/or sodium and lower contents of fiber, protein and micronutrients [33, 34]. Another finding was that more than half of the toddlers in our study (61.7%) consumed sugary drinks three times a week, with volumes of approximately 120 mL per day. These findings agree with what has been reported in other studies [35, 36].

The energy and macronutrient intakes on weekdays and weekend days among toddlers were adequate when considering the recommendations (e.g., energy 1000 kcal/d, proteins 10%-14%, CH 45%-65% and fats 30%-40%) [32, 37]. Likewise, the consumption levels of saturated fat and cholesterol were adequate [38, 39]. However, the consumption of added sugars (8 g/d) was excessive since consumption of these substances is not recommended for children under 24 months [40].

Regarding energy and nutrient intakes according to the type of feeding in the first semester of postnatal life among toddlers aged 12 to 24 months, we found that those who received FBF had lower energy consumption levels than those who received PBF and HMS. A possible explanation could be related to appetite regulation in breastfeeding infants due to the presence of regulatory hormones present in HM [41]. Another finding of interest was that toddlers who received HMS had higher intakes of energy, saturated fat, CH and sodium than those who received FBF or PBF. These findings are consistent with those reported by Chaparro [42], who found that infants who received BF during their first year of life had healthier dietary behaviors than children who received HMS.

### Adiposity in toddlers from 12–24 months

In the entire studied population, the adiposity values obtained by BIA (kg or percentage) were considered normal according to the reference of Butte [6]. Females had lower Z adiposity scores than males (*p* = 0.05). A possible explanation could be that in Mexico, malnutrition syndrome is more frequent in females, with important epidemiological and socio-anthropological implications, due to a potential discriminatory effect toward the female gender [43]. In contrast to this finding, it has been reported that females have more adiposity than males, which persists throughout life [44, 45].

There were no significant differences in adiposity according to the type of feeding that the studied toddlers received in the first semester of postnatal life. A possible explanation could be that changes in adiposity according to the type of feeding they received (e.g., FBF, PBF or HMS) occurred in a more significant manner in the first six months of life [11, 14]. Infants receiving BF appeared to have increased fat mass at 2, 4, and/or 6 months of age [11, 14], and BF is likely to protect infants with greater increases in fat mass in the first six months of life vs. infants who received HMS [11]. In addition, it has been stated that in the first months of life, higher fat mass could be part of an optimal phenotype influenced by BF, which could be related to protection against obesity development in later life stages [14, 15].

### Relationship between adiposity and feeding practices in the first two years of life

In the study sample, there was a direct relationship between adiposity and FBF duration. This could indicate that the longer the BF duration, the higher the adiposity in toddlers aged 12–24 months, which could be explained simply by changes in adiposity as toddler ages increase [8]. Alternatively, BF infants have been shown to have significant increases in fat mass accumulations during the first six months. However, these accumulations decrease or disappear after six months and tend to reverse at 12 months of age [8, 14, 44]. In contrast to this finding, an inverse relationship has been reported between BF duration and the indicators of adiposity and obesity [9, 10, 46–48]. This could be because breastfeeding plays a protective role against the development of obesity due to the presence of hormones in human milk that are responsible for regulating appetite [41].

In females, there was a direct relationship between adiposity and FBF duration, while in males, there was no significant difference. Similar to this finding, it has been reported that there are more associations among females between BF and variables related to adiposity [46]. It is not clear why this relationship is stronger in females than in males. However, one possible explanation is that the effects of hormones that are potentially involved in the metabolic effects of BF in infants could be influenced by sex [46].

We did not find any relationships between adiposity and age of CF introduction. This finding is consistent with that reported by Rodríguez [14] since they did not report a relationship between CF and fat mass. A possible explanation could be that the age of CF introduction adhered to the recommendations and that there were no early introductions (before four months). These findings reaffirm the behavior of avoiding early CF introduction because it is associated with higher adiposity and a higher risk of overweight or obesity in later life stages [49–52]. Interestingly, for females, there were inverse relationship between adiposity and the ages at which eggs, fish, and cheese were introduced. Proteins play a role in body composition, and it has been stated that high protein intake in childhood could increase the plasma and tissue concentrations of insulinogenic amino acids and the growth mediators of insulin and IGF-1, increasing adiposity [53].

In the entire studied sample and especially in males, there was relationship between adiposity and the age at which yogurt with added sugars was introduced. This finding emphasizes the importance of avoiding ultra-processed foods for different reasons during the first year of life; for example, the negative impact they have on adiposity, the development of obesity or other chronic-degenerative diseases and the development of inadequate food preferences and eating habits [54].

Another interesting finding was that we found a relationship between adiposity and the amount of added sugar consumed; therefore, this reaffirms that added sugars should be avoided in children under two years of age [40] because they increase the risk of obesity and other adverse health effects [54].

We found that infants who received HMS in the first semester of postnatal life exhibited various relationships between adiposity and the consumption of sugar-sweetened beverages, lipids, saturated fat, cholesterol and added sugars. Therefore, our study confirms that BF could contribute to the development of healthy eating behaviors, with a medium-term effect on food choices and food intake and consequently on adiposity [6, 9].

The main strength of this study was the analysis of adiposity using BIA since its determinations in the pediatric field have become very relevant due to the increasing prevalence of obesity in the early stages of life and its adverse effects on health. The second strength is that the age group we included (toddlers aged 12–24 months) because to date, there are few studies that include toddlers in analyses of adiposity by using BIA measurements. A limitation of this work was the cross-sectional study design because we could not analyze the changes in adiposity from 12 to 24 months of age and because we could not know the protective factors for obesity regarding feeding practices in the first two years of life. Another limitation was that in some cases, type II errors could occur since the group of toddlers that belonged to the HMS group was the smallest. Another limitation was that we did not assess maternal factors such as pre-pregnancy BMI, weight gain during pregnancy, or current maternal factors such as diet, physical activity, and their impact on infant feeding, which could help to understand the origins of obesity.

## Conclusions

Feeding practices in the first two years of life influence the formation of eating habits that contribute to lifelong eating habits and overall health. This study demonstrated that BF practices are improving in Mexico. However, a focus of attention could be the intake of sugary drinks and ultra-processed foods because they form part of toddler diets, and there was early introduction during CF in this group. The adiposity values were normal in this group of toddlers, and there were relationships between adiposity and duration of full breastfeeding, age of introduction of foods of animal origin and age of introduction of ultra-processed foods. Additionally, we demonstrated that breastfeeding contributes to adequate CF practices in toddlers. Future research with a longitudinal design is necessary to determine changes in adiposity and their relationship with feeding practices in the first two years of life, including other sociodemographic variables that could help us understand the variability of adiposity. In addition, future research could analyze VAT and SAT distributions in toddlers by using other measurement techniques to determine the effects of feeding practices in these two types of adipose tissue. 

## Data Availability

The databases from which the data for this manuscript were obtain can be found at the Institute of Human Nutrition of the University of Guadalajara. Email: inhu@cucs.udg.mx Phone + 523,336,189,667 and the datasets used and/or analysed during the current study available from the corresponding author on reasonable request.

## Abbreviations

BF: Breastfeeding
BIA: Bioelectrical impedance
BMI: Body mass index
CF: Complementary feeding
CH: Carbohydrates
EBF: Exclusive breastfeeding
FBF: Full breastfeeding
g: Grams
g/d: Grams per day
HMS: Human milk substitutes
kcal/d: Kcal per day
NS: Not significant
n: Number of participants
PBF: Partial breastfeeding
PrBF: Predominant breastfeeding
Q: Quartile
r: Correlation coefficient
R^2^: Adjusted coefficient of determination
SD: Standard deviation
X: Average
